# How a universal health system reduces inequalities: lessons from England

**DOI:** 10.1136/jech-2015-206742

**Published:** 2016-01-19

**Authors:** Miqdad Asaria, Shehzad Ali, Tim Doran, Brian Ferguson, Robert Fleetcroft, Maria Goddard, Peter Goldblatt, Mauro Laudicella, Rosalind Raine, Richard Cookson

**Affiliations:** 1 Centre for Health Economics, University of York, York, UK; 2 Department of Health Sciences, University of York, York, UK; 3 Public Health England, York, UK; 4 Norwich Medical School, University of East Anglia, Norwich, UK; 5 Institute of Health Equity, University College London, London, UK; 6 School of Health Sciences, City University London, London, UK; 7 University College London, London, UK

**Keywords:** Health inequalities, ACCESS TO HLTH CARE, PRIMARY CARE

## Abstract

**Background:**

Provision of universal coverage is essential for achieving equity in healthcare, but inequalities still exist in universal healthcare systems. Between 2004/2005 and 2011/2012, the National Health Service (NHS) in England, which has provided universal coverage since 1948, made sustained efforts to reduce health inequalities by strengthening primary care. We provide the first comprehensive assessment of trends in socioeconomic inequalities of primary care access, quality and outcomes during this period.

**Methods:**

Whole-population small area longitudinal study based on 32 482 neighbourhoods of approximately 1500 people in England from 2004/2005 to 2011/2012. We measured slope indices of inequality in four indicators: (1) patients per family doctor, (2) primary care quality, (3) preventable emergency hospital admissions and (4) mortality from conditions considered amenable to healthcare.

**Results:**

Between 2004/2005 and 2011/2012, there were larger absolute improvements on all indicators in more-deprived neighbourhoods. The modelled gap between the most-deprived and least-deprived neighbourhoods in England decreased by: 193 patients per family doctor (95% CI 173 to 213), 3.29 percentage points of primary care quality (3.13 to 3.45), 0.42 preventable hospitalisations per 1000 people (0.29 to 0.55) and 0.23 amenable deaths per 1000 people (0.15 to 0.31). By 2011/2012, inequalities in primary care supply and quality were almost eliminated, but socioeconomic inequality was still associated with 158 396 preventable hospitalisations and 37 983 deaths amenable to healthcare.

**Conclusions:**

Between 2004/2005 and 2011/2012, the NHS succeeded in substantially reducing socioeconomic inequalities in primary care access and quality, but made only modest reductions in healthcare outcome inequalities.

## Introduction

Equity is widely accepted by the medical professions as a fundamental element of quality,^1^ ^2^ and providing equitable care is a priority for most national healthcare systems.^3^ Provision of universal coverage is a necessary, but not sufficient, requirement for achieving this goal. In the USA, the Patient Protection and Affordable Care Act aims to provide near-universal access to healthcare coverage and to improve quality and value.^4^ Recent state-level expansions of healthcare coverage have improved access to care for disadvantaged populations,^5^ and have been associated with improvements in mortality for causes amenable to healthcare.^6^ However, failure to address inequalities in care within the covered population will ultimately undermine wider programmes to improve quality of care and patient outcomes.^7^

In the UK, the National Health Service (NHS) has provided universal, comprehensive healthcare free at the point of delivery since 1948. Despite this, there are clear inequities in healthcare in the UK, and poorer access and worse patient outcomes remain strongly associated with social disadvantage.^8^ ^9^ Recognising this, in 2003 the UK Government made reducing health inequality a priority for the NHS in England, as part of a cross-governmental strategy with explicit national targets for reducing health inequality by 2010^10^—the world's first national strategy of this kind.^11^ Strengthening primary care was central to these efforts, which included: (1) major investments in primary care supply and quality from 2004, including the world's largest primary care pay-for-performance programme^12^; (2) targeted investment in primary care supply in under-doctored areas of the country from 2008^13^ and (3) national guidance and support for effective primary care interventions for chronic conditions in disadvantaged adults from 2007 to 2009.^14^

It is not known how far the NHS contributed to reducing health inequalities during this key period because socioeconomic inequalities in primary care access, quality and outcomes have not been routinely monitored.^15^ This hampers efforts to improve equity, since what is not measured may be marginalised.^16^ National health inequality targets introduced in the 2000s were limited from a healthcare quality perspective as they are related to local government areas, thus masking important inequalities within these areas. They also focused on life expectancy and infant mortality, over which healthcare providers have little direct control since they are strongly influenced by social and economic factors (eg, living and working conditions), and related lifestyle behaviours (eg, smoking, diet and exercise).

In this paper, we address these weaknesses by constructing a suite of four key summary measures relating to trends in socioeconomic inequality in healthcare access, quality and outcomes for which the healthcare system can plausibly be held to account. We present data describing trends in absolute as well as relative inequality in these indicators at small area level, and provide the first comprehensive assessment of trends in healthcare equity performance during a key period of sustained effort by a national healthcare system to reduce socioeconomic inequalities in primary care access, quality and outcomes.

## Methods

### Data sources

We extracted health data from four national administrative databases for financial years 2004/2005 to 2011/2012: (1) the annual NHS General and Personal Medical Services workforce census (physician supply); (2) the Quality and Outcomes Framework (QOF)—the national primary care pay-for-performance programme (primary care quality); (3) hospital episode statistics (hospital admissions) and (4) the Office for National Statistics (mortality). Data on physician supply and primary care quality were attributed from practice level to small area level using the NHS Attribution Data Set of GP-registered populations. Data on hospital activity and mortality were aggregated to small area level from individual level.

The basic geographical unit of analysis was the 2001 ‘lower super output area’ (LSOA). There are 32 482 of these small area neighbourhoods, covering approximately 1500 people each (minimum 1000 and maximum 3000). We measured the population size of each neighbourhood by age–sex group using mid-year population estimates from the Office for National Statistics (ONS) for years 2004–2011. We measured the socioeconomic status of each neighbourhood using the index of multiple deprivation (IMD 2010).

### Indicators

We aimed to provide a comprehensive assessment of socioeconomic inequalities of primary care access, quality and outcomes for which the NHS can be held accountable in its efforts to tackle health inequality. The indicator selection process included: reviewing existing indicators used by the NHS to monitor healthcare performance; consulting with health indicator experts about technical feasibility, and with clinical and policy experts about clinical and policy relevance; and a small-scale public consultation exercise. Four key indicators were selected:

1. Primary care supply

We defined primary care supply as patients per full-time equivalent (FTE) general practitioner (GP), excluding registrars and retainers. In line with previous studies, we focused on FTE GP principals and salaried GPs, who make up the vast majority of the workforce.^17^ Neighbourhood populations were adjusted for their relative needs for primary care using the workload adjustment aspect of the Carr-Hill formula for primary care resource allocation.^18^ This adjustment takes into consideration the age and sex structure and IMD 2010 ‘health deprivation and disability’ score of each LSOA.

- 2. Primary care quality

We defined primary care quality using a modified version of the QOF-based public health impact score proposed by Ashworth *et al*.^19^ Our indicator is a score between 0 and 100 calculated as a weighted average of clinical process quality from 16 QOF indicators that were collected on a consistent basis throughout our study period. Each of these QOF indicators measures the percentage of the relevant patient population achieving a particular clinical quality target. Weights used to combine these indicators into an overall score were proportional to their relative importance in terms of the estimated mortality reduction impact associated with improvement on the indicator. We measured practice-reported performance, which excludes patients reported as ‘exceptions’ (and therefore considered not to be appropriate for the quality targets).^20^ In sensitivity analysis we included exception reported patients (see online supplementary appendix 3 for details).

- 3. Preventable hospitalisation

We defined preventable hospitalisation as the proportion of people with an emergency admission for a chronic ambulatory care sensitive condition—admissions that are potentially avoidable if these chronic conditions are appropriately managed in primary care—examples of such hospital admissions are those associated with asthma and diabetes.^21^ We focused on chronic rather than acute ambulatory care sensitive conditions, as the former are likely to be more sensitive to changes in primary care supply and quality. We used the same list of chronic ambulatory care sensitive conditions as the NHS Outcomes Framework (Indicator 2.3i).^22^ We indirectly standardised each year of data for age and sex at LSOA level.

- 4. Amenable mortality

We defined amenable mortality as the proportion of people dying from causes considered amenable to healthcare. We used the list of causes of death and age ranges where deaths from these causes are considered amenable to healthcare from the NHS Outcomes Framework (Indicator 1.1).^23^ As with preventable hospitalisation, we indirectly standardised amenable mortality for age and sex at LSOA level.

The two healthcare outcome indicators are widely used, internationally, to monitor the performance of whole healthcare systems, and are particularly useful for monitoring the performance of primary care and the coordination of care between primary and secondary services.^24^ ^25^ Full details of the indicator definitions and the standardisation processes are provided in online supplementary appendices 1 and 2, respectively.

### Analysis

Our primary measures of inequality were the slope index of inequality (SII) and relative index of inequality (RII), based on linear regression analysis at LSOA level. Each indicator was modelled as a linear function of LSOA level deprivation, entered as a continuous variable scaled from 0 to 1. The SII is the coefficient in this regression; the RII is that coefficient divided by the mean. The SII can be interpreted as the modelled absolute gap between the most and least-deprived small area, allowing for the whole socioeconomic gradient; the RII can be interpreted as the proportionate gap relative to the average. Alongside these quantitative measures we also visualised the relationship between deprivation and inequality graphically to aid in the understanding and interpretation of these measures.

We also computed the ‘inequity gap’, based on a counterfactual situation of full equality in which all neighbourhoods do as well as the least-deprived neighbourhood in terms of modelled achievement on the indicator. For primary care supply, the ‘inequity gap’ is calculated as the number of additional physicians required to achieve full equality. For primary care quality, it is the average deficit in quality attributable to socioeconomic inequality. For rates of preventable hospitalisation and amenable mortality it is the number of avoidable hospitalisations and deaths attributable to socioeconomic inequality.

Linear regression models were computed using pooled data for the first and last years, including interaction terms between year and deprivation to determine the magnitude of—and test for the statistical significance of—changes in inequality between the beginning and end of the analysis period.

## Results

### Inequalities in 2004/2005

**Figure 1:**
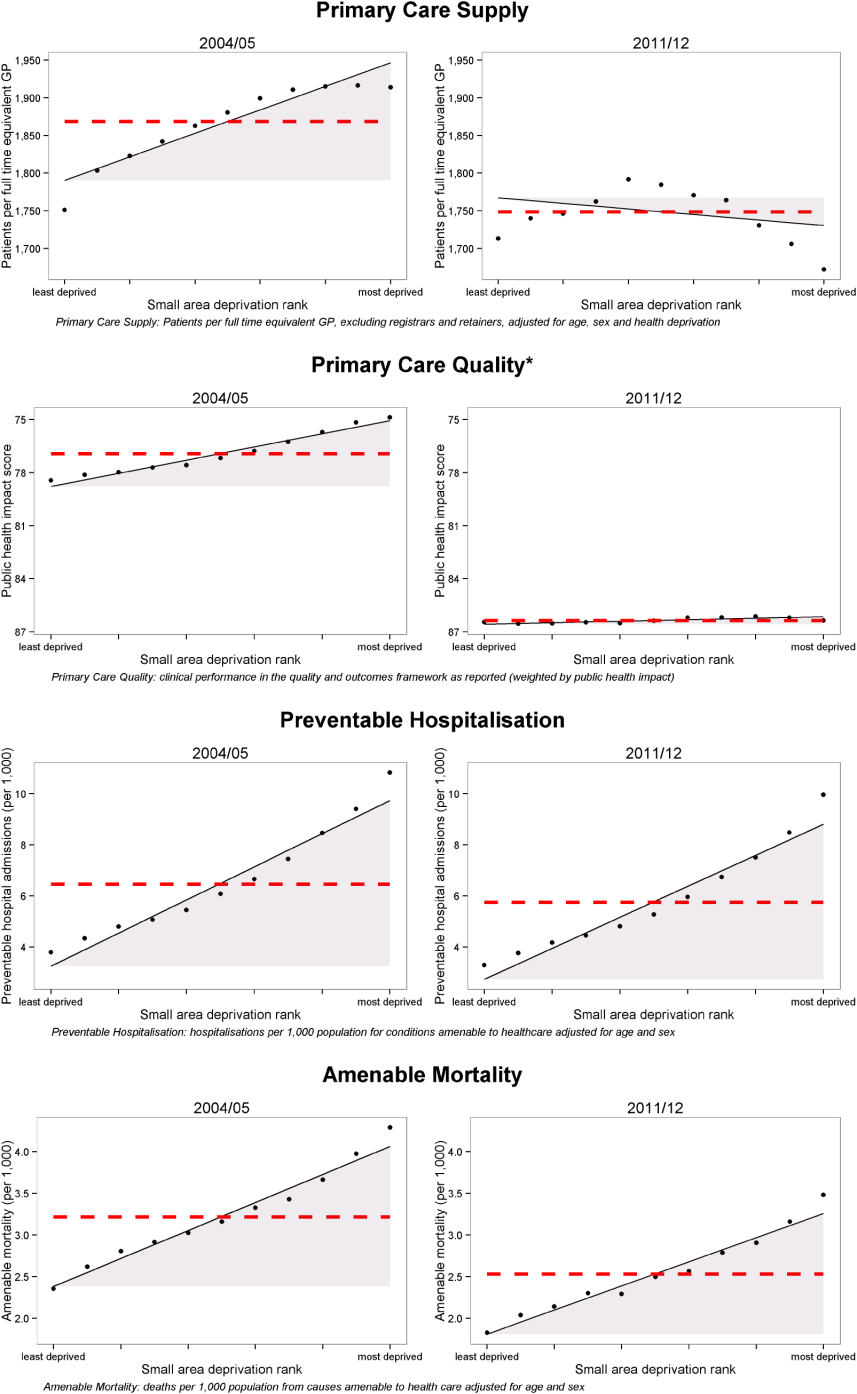
Scatter plots of indicators in 2004/2005 and 2011/2012. The black dots show deprivation decile groups of neighbourhoods (approximately 3200 neighbourhoods per dot); the solid black line shows a linear regression through all 32 482 neighbourhoods; the shaded area shows the inequality gap; and the dashed red line shows the national average level for the indicator. *Inverted axis on primary care quality to ease comparisons with other indicators, where decreasing implies improvement (GP, general practitioner).

There were clear and substantial socioeconomic gradients in all four indicators in 2004/2005 (figure 1), with less favourable primary care provision and health outcomes in more-deprived areas. For primary care supply, there were fewer GPs relative to measured need (and therefore, more patients per GP) in deprived neighbourhoods than in less-deprived neighbourhoods. This socioeconomic inequality was associated with a deficit of 1008 GPs (924 to 1093) nationally (table 1). In other words, equalising GP provision in all neighbourhoods to the modelled level of GP provision in the least-deprived neighbourhood would require an additional 1008 GPs in relatively deprived neighbourhoods. Socioeconomic inequality was also associated with a deficit of 1.86 percentage points (1.79 to 1.94) in primary care quality, 160 397 (158 090 to 162 703) preventable hospitalisations, and 41 433 (39 899 to 42 966) amenable deaths.

**Table 1:** Socioeconomic healthcare inequalities in England, comparing 2004/2005 with 2011/2012

Indicator	England mean (95% CI)	RII (95% CI)	SII (95% CI)	Inequality gap (95% CI)
Primary care supply				
2004	1814 (1814 to 1814)	0.09 (0.08 to 0.09)	156.1 (141.29 to 170.91)	1008 (924 to 1093)
2011	1689 (1689 to 1689)	–0.02 (−0.03 to −0.01)	–36.61 (−49.8 to −23.42)	–335 (−436 to −233)
Change 2011–2004	–125 (−125 to −125)	–0.11 (−0.12 to −0.1)	–192.71 (−212.55 to −172.87)	–1343 (−1473 to −1213)
Primary care quality				
2004	76.91 (76.91 to 76.91)	0.05 (0.05 to 0.05)	3.73 (3.58 to 3.87)	1.86 (1.79 to 1.94)
2011	86.34 (86.34 to 86.34)	0.01 (0.00 to 0.01)	0.44 (0.37 to 0.51)	0.22 (0.18 to 0.26)
Change 2011–2004	9.44 (9.44 to 9.44)	–0.04 (−0.05 to −0.04)	–3.29 (−3.45 to −3.13)	–1.64 (−1.72 to −1.56)
Preventable hospitalisation				
2004	6.43 (6.43 to 6.44)	1.01 (0.99 to 1.02)	6.48 (6.39 to 6.58)	160 397 (158 090 to 162 703)
2011	5.73 (5.73 to 5.74)	1.06 (1.04 to 1.07)	6.07 (5.97 to 6.16)	158 396 (155 995 to 160 797)
Change 2011–2004	–0.7 (−0.71 to −0.69)	0.05 (0.03 to 0.07)	–0.42 (−0.55 to −0.29)	–2000 (−5270 to 1284)
Amenable mortality				
2004	3.21 (3.21 to 3.22)	0.52 (0.5 to 0.54)	1.68 (1.62 to 1.74)	41 433 (39 899 to 42 966)
2011	2.53 (2.53 to 2.54)	0.57 (0.55 to 0.59)	1.45 (1.4 to 1.5)	37 983 (36 552 to 39 415)
Change 2011–2004	–0.68 (−0.69 to −0.67)	0.05 (0.02 to 0.08)	–0.23 (−0.31 to −0.15)	–3449 (−5516 to −1375)

The England means and the SII indices are measured in terms of patients per physician, average primary care quality, preventable hospitalisation per 1000, and amenable mortality per 1000. The RII indices are the SII indices as a proportion of the England means. The inequality gaps refer to the number of GPs required to eliminate inequality, the average quality loss attributable to inequality, the total excess hospitalisations attributable to inequality, and the total excess mortality attributable to inequality.

GP, general practitioners; RII, relative index of inequality; SII, slope index of inequality.

### Changes in inequality between 2004/2005 and 2011/2012

All four indicators improved on average (ie, inequalities reduced) between 2004/2005 and 2011/2012. Inequalities in primary care supply and quality decreased substantially, to the extent of being virtually eliminated by the end of the period, whereas changes in the social gradient in preventable hospitalisation and amenable mortality were less pronounced (figure 1). By 2011/2012, the numbers of GPs had increased in all areas, with the greatest increases in the most-deprived areas, leaving neighbourhoods in the middle of the deprivation range with the fewest GPs per patient. Socioeconomic inequality had been reduced to such an extent that deprived neighbourhoods had slightly more GPs relative to need than less-deprived neighbourhoods, and socioeconomic inequality was associated with a surplus of 335 GPs (233 to 436), that is, equalising GP provision in all neighbourhoods to the level of the least-deprived neighbourhood would require losing 335 GPs from relatively deprived neighbourhoods.

**Figure 2:**
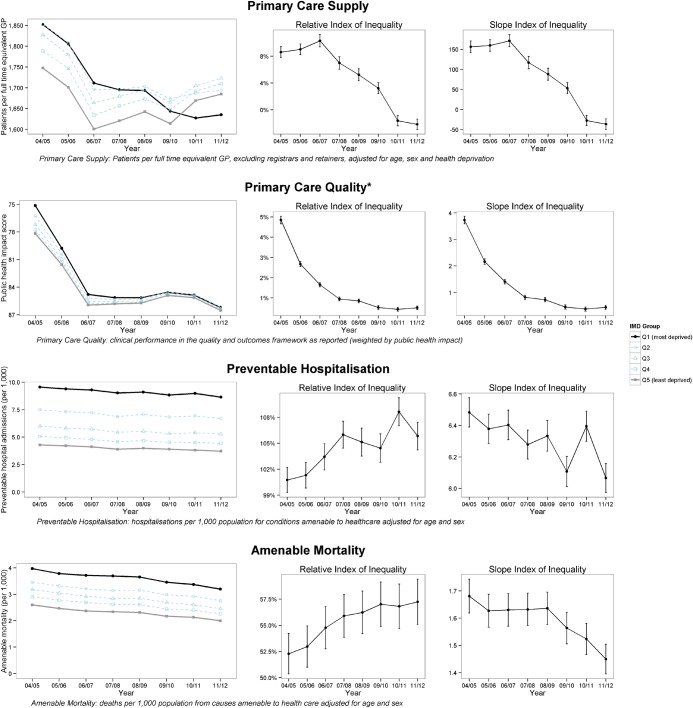
Inequality trends from 2004/2005 to 2011/2012. *Inverted axis on primary care quality to ease comparisons with other indicators, where decreasing implies improvement (GP, general practitioner; IMD, index of multiple deprivation).

By 2011/2012, socioeconomic inequality was also associated with an average deficit in primary care quality of 0.22 percentage points (0.18 to 0.26), 158 396 excess preventable hospitalisations (155 995 to 160 797), and 37 983 excess amenable deaths (36 552 to 39 415). Looking more closely at the trends in inequality in the indicators over the period (table 1 and figure 2) there is a clear trend of decreasing inequality in both absolute and relative terms for both primary care supply and primary care quality. By contrast, preventable hospitalisation and amenable mortality show a mixed pattern of decreasing absolute inequality but increasing relative inequality.

## Discussion

Our study presents the first comprehensive national picture of how far the NHS in England succeeded in reducing socioeconomic inequalities in primary care supply, quality and outcomes from 2004/2005 to 2011/2012. During this period, primary care supply, quality and outcomes for the average patient all improved. We find that socioeconomic inequalities in both primary care supply relative to need and primary care quality decreased substantially in absolute and relative terms. By the end of the period, inequality in primary care supply had been eliminated, and inequality in primary care quality had been nearly eliminated. By contrast, inequality trends in preventable hospitalisation and amenable mortality were mixed, showing decreasing absolute inequality but increasing relative inequality. By 2011/2012, deprived neighbourhoods had slightly better primary care supply than less-deprived neighbourhoods (relative inequality –2%), and only slightly worse primary care quality (relative inequality 1%). However, there remained large inequalities in preventable hospitalisation (relative inequality 106%) and amenable mortality (relative inequality 57%).

### Strengths and weaknesses of the study

We used data on the entire population of England, including workload and quality data on virtually all primary care practices in England, and outcomes data on virtually all individuals in England. We used comprehensive indicators spanning the entire range of activities of the healthcare system, and inequality measures based on the entire socioeconomic gradient across all 32 482 small areas of England. We examined inequality in absolute and relative terms, because absolute and relative inequality can change in opposite directions when the mean is changing over time.^26^ One of our measures—the RII—can also be compared between indicators measured on different scales to help assess the relative magnitude of different kinds of inequality.

However, our study does not include data on privately funded healthcare, which accounts for approximately 15% of total health expenditure in the UK.^27^ We also lack detailed national data on changing patterns of multimorbidity at small area level. One consequence is that our study may underestimate additional needs for primary care in deprived neighbourhoods, which are likely to suffer from a greater burden of multimorbidity.^28^ We also cannot assess how far observed trends in preventable hospitalisation and amenable mortality are due to trends in multimorbidity outside the control of the NHS. Another limitation is that the administrative health data sets do not contain information on individual socioeconomic characteristics. We therefore used the IMD, which assumes that individuals generally conform to the socioeconomic profile of their residential neighbourhood. Finally, our measure of primary care quality is based on indicators drawn from the UK primary care pay-for-performance scheme, which only captures part of clinical practice.^29^ Under this scheme, improvements in quality were most rapid in practices with low baseline performance, and these practices were concentrated in more-deprived areas.^30^ It is possible that aspects of primary care quality that were not financially incentivised and monitored did not follow the same pattern, and inequalities in these may have persisted or even widened.

### Findings

The NHS succeeded in reducing inequality in primary care supply and quality from 2004/2005 to 2011/2012, eliminating the inequity in primary care supply and almost eliminating the inequity in primary care quality. These changes can partly be attributed to the substantial investments in primary care in the mid-2000s to late 2000s, including the QOF pay-for-performance programme from 2004/2005, and provision of additional funding for new GP practices in ‘under-doctored’ areas of the country from 2006.^13^ ^31^ However, the NHS did not have comparable success in reducing socioeconomic inequalities in healthcare outcomes. Although absolute inequalities in healthcare outcomes decreased slightly from 2004/2005 to 2011/2012, relative inequalities increased, and substantial inequalities remained in 2011/2012 in preventable hospitalisation and amenable mortality. While not wholly unexpected,^32^ ^33^ this is still perhaps disappointing, given that this was a period of sustained large-scale expenditure growth in the NHS in England,^34^ and that tackling health inequality was a high priority for the NHS.^35^ It is possible that non-NHS factors were acting to increase socioeconomic inequalities in healthcare outcomes during this period—evidence suggests that socioeconomic inequalities increased between 2003 and 2008 for smoking, poor diet, physical inactivity and other unhealthy behaviours.^36^ It is also possible that changes in primary care supply and quality have not yet been given sufficient time to substantially reduce inequalities in healthcare outcomes, or that the national pay-for-performance programme overemphasised management of existing chronic diseases over primary prevention.

### Comparison with other studies

One previous national study examined socioeconomic inequality in preventable hospitalisation in England covering years 2001/2002–2012/2013.^37^ This study finds similar trends to those we observe, showing a gradual decrease in the rate of chronic ambulatory care sensitive emergency admissions for the average patient, and substantial and persistent socioeconomic inequalities in ambulatory care sensitive emergency admissions over the period. One previous national study examined socioeconomic trends in amenable mortality^38^ in England from 2001/2002 to 2011/2012. However, this study was conducted at a large area level (324 local authorities) which may potentially mask changing patterns of inequality within these large areas, and it excluded mortality in people aged over 75 years. This study found both average levels and absolute measures of inequality in amenable mortality to have fallen over this period. Our finer grained analysis looking at much smaller areas (32 482 LSOAs) and following the ONS definition of amenable mortality, hence also including mortality for certain conditions such as HIV/AIDS and injuries in those over 75 years of age, confirms this basic pattern, though revealing a widening of relative inequality that was not apparent in the previous study. Furthermore, our inclusion of this older section of the population results in a higher overall rate of amenable mortality, and the more detailed level of analysis we employ reveals wider socioeconomic inequalities.

## Conclusion

Reducing inequality in healthcare outcomes is more complex and challenging than reducing inequality of access to healthcare.^39^ Socioeconomic inequalities in preventable hospitalisation and amenable mortality are not only due to inequalities in the supply of primary and hospital care. They are also attributable to socioeconomic-related differences in, and complex interactions between (1) multimorbidity; (2) patient behaviours including healthcare seeking, self-care and lifestyle; (3) informal social support networks; (4) social care supply and quality; (5) primary care provider behaviour; (6) secondary care provider behaviour and (7) the coordination of care between primary, secondary and social care providers. Reducing socioeconomic inequalities in healthcare outcomes is therefore likely to require complex interventions to improve the coordination of care between multiple actors within and outwith the healthcare system. There is a growing body of evidence about effective interventions to reduce preventable hospitalisation and amenable mortality, but little is known about how to reduce socioeconomic inequalities in these healthcare outcomes.^40^ ^41^ It is our hope that the indicators developed in this study can play a role in helping to develop the evidence base for reducing inequalities in healthcare outcomes through application to equity monitoring at local, national and international levels.

**What is already known on this subject**

- There are socioeconomic inequalities in primary care access, quality and outcomes even in high-income countries with universal healthcare systems.
- Reducing these inequalities by strengthening primary care was a key priority for the National Health Service (NHS) in England from 2004/2005 to 2011/2012, as part of the world's first cross-government strategy for reducing health inequality.
- It is not known how far the NHS succeeded in addressing this priority, since national trends in healthcare equity are still not routinely monitored.

**What this study adds**

- This study presents the first comprehensive assessment of national trends in socioeconomic inequalities in primary care access, quality and outcomes in England from 2004/2005 to 2011/2012.
- During this period, there were substantial reductions in socioeconomic inequalities in primary care supply and quality, but only modest reductions in preventable emergency hospitalisation and mortality amenable to healthcare.
- We have developed a suite of indicators that could be used in other countries to monitor the contribution of healthcare services to tackling wider inequalities in community health.

## Supplementary Material

Web appendix 1

Web appendix 2

Web appendix 3
