# Supplementary material for: How a universal health system reduces inequalities: lessons from England
Source: J Epidemiol Community Health. 2016 Jan 19;70(7):637–43. doi: 10.1136/jech-2015-206742 (PMC4941190; doi:10.1136/jech-2015-206742)
Supplement: Web appendix 1 [file jech-2015-206742-s1.pdf]

How a universal health system reduces inequalities: lessons from England

Appendix 1: Indicator Definitions

Primary care supply

Definition:

Primary care supply is defined as the number of patients per full time equivalent GP, excluding registrars and retainers, adjusted for age, sex and neighbourhood ill-health using the Carr-Hill workload adjustment. This version of the formula was recommended in 2007 by the Formula Review Group established by NHS Employers and the BMA, and though never implemented in practice it remains the most authoritative and up-to-date analysis of the determinants of primary care workload in England.

The numerator is the total number of people alive at the mid-point of the current financial year. In practice, we use ONS mid-year estimates of the population during the middle of the calendar year. The denominator is the number of FTE GPs excluding registrars and retainers at the mid-point of the current financial year attributed to each small area.

Technical details:

Our data on primary care supply at GP practice level were obtained from the annual National Health Service General and Personal Medical Services workforce census, taken at 30th September each year, midway through the financial year. In keeping with standard measures of the GP workforce we exclude GP registrars and GP retainers from our measure.

We used this data to construct a whole-population national data set at small area (LSOA) level by using the NHS Attribution Data Set of GP-registered populations to attribute FTE GPs from GP practices to LSOAs. The attribution dataset details the LSOAs in which the patients registered with the practice live. We use this information to determine the proportion of the FTE GP workforce attached to the practice to attribute to each of the LSOAs that the patients registered with the practice live in. Applying this attribution calculation to each GP practice and then aggregating the GP supply attributed from the different practices at LSOA level gives us our measure of primary care supply at LSOA level. We linked practice level data on primary care supply for the ten years 2004/05 through 2011/12 with corresponding LSOA level data on population and deprivation. We use data from all 9,092 general practices in the English NHS that were open for at least one year of the study period.

We then need-weighted the population for each small area for age, sex and IMD 2010 health domain using the Carr-Hill formula workload adjustment (updated 2007 version) to upscale populations that are expected to require more primary care and downscale populations expected to require less (Formula Review Group 2007; Hippisley-Cox et al

2006). The “Carr-Hill” formula is used for distributing funding to GP practices. We do not adjust for temporary resident population, the fourth and final workload adjustment factor in the Carr-Hill formula, as the HSCIC were unable to provide us with the patient level data necessary to make this adjustment.

Primary care quality

Definition:

Primary care quality is a score between 0 and 100 defined as a weighted average of clinical process quality from 16 indicators in the national quality and outcomes framework (QOF). Each indicator measures the percentage of the relevant patient population for whom the quality target is achieved. The weights used to combine these indicators into a primary care quality score are proportional to importance of the individual indicators in terms of the estimated mortality reduction impact associated with improvement on the indicator.

Technical details:

GP practices record the number of patients with each condition who are listed in their practice registers. For each clinical indicator, the number of patients deemed appropriate for that indicator is the denominator and the number of patients for whom the indicator was met is the numerator. The reported achievement on the indicator is the percentage of relevant patients for whom the practice met the indicator quality target.

We started with a group of 20 QOF indicators identified by Ashworth et al (2013) based on available evidence on mortality reduction. We then selected 16 out of the 20 indicators for which data were available throughout our period of analysis in a consistent format. Each indicator was then weighted based on importance in terms of the estimated number of lives saved per 100,000 patients. These weights were derived from Ashworth et al (2013) who identified the highest level of evidence for risk reduction in all-cause mortality and converted risk reduction estimates into estimated mortality reduction rates per 100,000 population per annum (see table A.1.1 for details).

Numerators and denominators for the QOF indicators were attributed from GP practice to LSOA level in an identical manner to that used to attribute primary care supply as described above. The QOF indicators were then calculated at LSOA level and these were then combined using the weighting process described to give average performance in terms of primary care quality score at LSOA level.

We did not need to standardise this indicator, since it is a nationally comparable performance measure that already allows for case mix and other characteristics of the GP practice population. Factors such as the age, sex and disease prevalence of the GP practice population are not legitimate justifications for variation in GP performance on these measures.

Table A.1.1: List of conditions in the Quality and Outcomes Framework (QOF)

QOF indicator	Summary description of indicator	Crude prevalence per 100,000 registered patients, mean (SD)	Annual mortality reduction, per 100,000 registered patients
DM18	Diabetes: influenza vaccination	4420 (1881)	63.7
CHD12	CHD: influenza vaccination	3448 (1487)	61.6
BP5a	Hypertension: BP ≤150/90 mmHg	13 548 (5117)	48.2
CHD10a	CHD: beta-blocker treatment	3448 (1487)	45.9
STROKE10	Stroke/TIA: influenza vaccination	1649 (967)	28.1
DM23a	Diabetes: HbA1c ≤7.0%	4420 (1881)	26.5
COPD8	COPD: influenza vaccination	1626 (958)	24.9
CHD9a	CHD: aspirin or other antithrombotic therapy	3448 (1487)	24.8
CHD8a	CHD: cholesterol ≤5.0 mmol/l	3448 (1487)	15.8
STROKE12a	Stroke (non-haemorrhagic): aspirin or other antithrombotic therapy	1080 (649)	15.8
DM12	Diabetes: BP ≤145/85 mmHg	4420 (1881)	13.5
CHD6a	CHD: BP ≤150/90 mmHg	3448 (1487)	11.3
SMOKING4	CHD, stroke/TIA, hypertension, DM, CKD, COPD, asthma, psychosis: smoking cessation advice	3903 (2525)	10.9
DM25	Diabetes: HbA1c ≤9.0%	4420 (1881)	7.4
DM15a	Diabetes with proteinuria or microalbuminuria: ACEI or ARB therapy	505 (513)	3.4
CHD11a	CHD (myocardial infarction): ACEI or ARB therapy	572 (291)	1.5

Preventable hospitalisation

Definition:

Preventable hospitalisation is defined as the number of people per 1,000 population having one or more emergency hospitalisations for a chronic ambulatory care sensitive condition, adjusting for age and sex.

The numerator is the number of people with emergency hospital admissions (both finished and unfinished admission episodes, excluding transfers) for specific long-term conditions which should not normally require hospitalisation. This is derived from the Hospital Episode Statistics (HES) Admitted Patient Care (APC), provided by the Health and Social Care Information Centre (HSCIC).

The denominator is the total number of people alive at mid-point in the current financial year. The Office for National Statistics (ONS) mid-year England population estimates for the respective calendar years are used for this purpose.

Technical details:

This indicator measures the number of people having an emergency hospital admission per 1,000 of population for specific long-term conditions considered amenable to health care. This is often used as an indicator of the performance of primary care and the interface between primary and secondary care. We use the list of conditions defined in the NHS outcomes framework indicator 2.3i (see Table A.1.2 below). Hospital admissions for all ages, including young children and people over 75, are included in this indicator.

We calculate indirectly standardised emergency hospital admission rate for each small area to allow for differing age and sex structure by deprivation level. To do so, we start with individual level HES data on emergency admissions and aggregate up to small area level. We then compute the expected hospitalisation counts for each small area by applying national age-sex hospitalisation rates to small area level numbers of people in each age-sex group. We then compute the adjusted rate for each small area as the product of the ratio of observed over expected count for the small area and the national rate. We then compute the adjusted count for each small area as adjusted rate times the small area population. Finally, we aggregate up this adjusted count to quantile group level to present adjusted count per 1,000 people in each quantile group. The calculations are presented in Appendix 2.

Figure A.1.1 shows trends in preventable hospitalisation for each quintile group by age and sex.

Figure A.1.1: Trends in preventable hospitalisation (fixed x-axis for age group comparisons, then floating x-axis for deprivation quintile group comparisons)

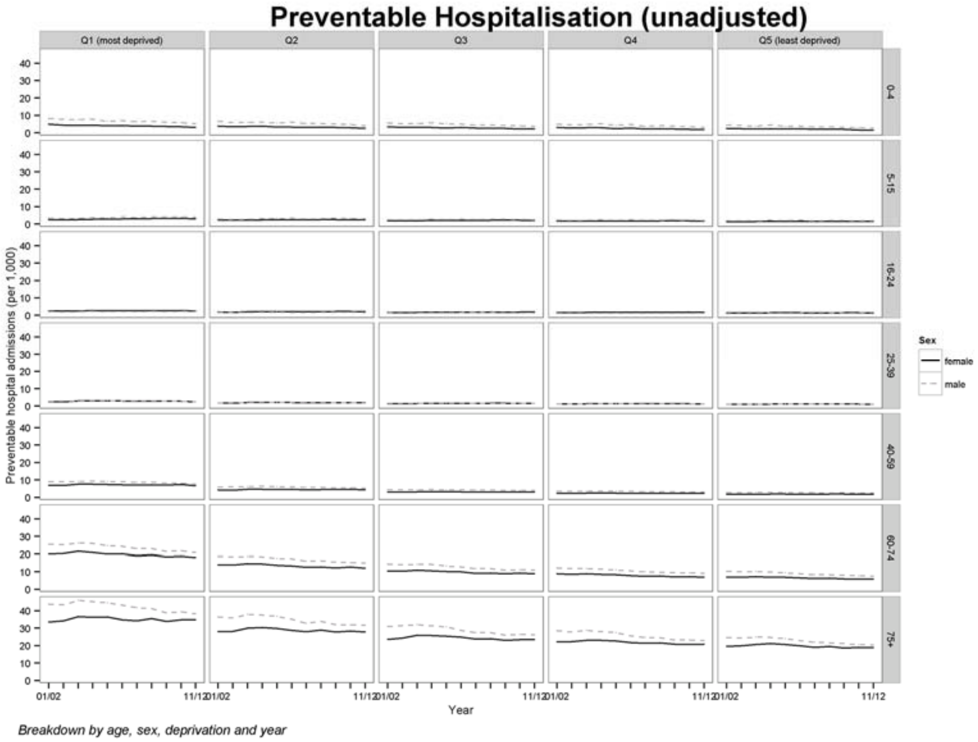

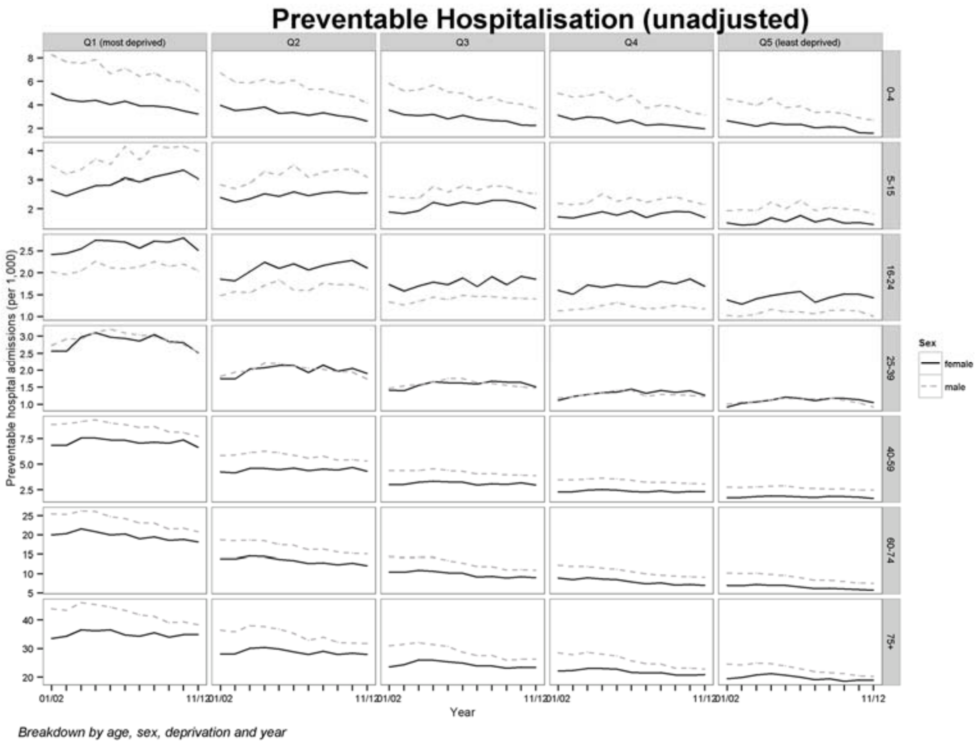

Table A.1.2: ICD-10 codes for chronic ambulatory care sensitive conditions (Health and Social Care Information Centre 2014)
This is based on a list produced by the ONS and adopted by the NHS Outcomes Framework.

**Infections**	
B18.1	Chronic viral hepatitis B without delta-agent
B18.0	Chronic viral hepatitis B with delta-agent
**Nutritional, endocrine and metabolic**	
E10	Insulin-dependent diabetes mellitus
E11	Non-insulin-dependent diabetes mellitus
E12	Malnutrition-related diabetes mellitus
E13	Other specified diabetes mellitus
E14	Unspecified diabetes mellitus
**Diseases of the blood**	
D50.1	Sideropenic dysphagia
D50.8	Other iron deficiency anaemias
D50.9	Iron deficiency anaemia, unspecified
D51	Vitamin B12 deficiency anaemia
D52	Folate deficiency anaemia
**Mental and behavioural disorders**	
F00	Dementia in Alzheimer disease
F01	Vascular dementia
F02	Dementia in other diseases classified elsewhere
F03	Unspecified dementia
**Neurological disorders**	
G40	Epilepsy
G41	Status epilepticus
**Cardiovascular diseases**	
I10X	Essential (primary) hypertension
I11.0	Hypertensive heart disease with (congestive) heart failure
I11.9	Hypertensive heart disease without (congestive) heart failure
I13.0	Hypertensive heart and renal disease with (congestive) heart failure
I20	Angina pectoris
I25	Chronic ischaemic heart disease
I50	Heart failure
I48X	Atrial fibrillation and flutter
J81X	Pulmonary oedema
**Respiratory diseases**	
J20	Acute bronchitis
J41	Simple and mucopurulent chronic bronchitis
J42X	Unspecified chronic bronchitis
J43	Emphysema
J44	Other chronic obstructive pulmonary disease
J45	Asthma
J46X	Status asthmaticus
J47X	Bronchiectasis

Amenable mortality

Definition:

Amenable mortality is defined as the number of deaths per 1,000 people from causes considered amenable to healthcare, allowing for age and sex. The numerator is the number of people who died in the current financial year due to a cause of death considered amenable to health care. The denominator is the total number of people alive at mid-point in the current financial year.

Technical details:

Amenable mortality was defined according to the conditions listed in the ONS Outcomes Framework (see table A.1.3). This includes conditions that are responsible for at least 100 deaths in a year and that have a clear link between the number of deaths and healthcare interventions. The classification takes account of appropriate age limits and each death is counted only once.

We calculate indirectly standardised amenable mortality rate for each small area to allow for differing age and sex structure by deprivation level. To do so, we start with individual-level ONS mortality data and aggregate up to small area level. We then compute the expected number of deaths in each small area by applying national age-sex mortality rates to small area level numbers of people in each age-sex group. We then compute the adjusted rate for each small area as the product of the ratio of observed over expected count for the small area and the national rate. We then compute the adjusted count for each small area as adjusted rate times the small area population. Finally, we aggregate up this adjusted count to quantile group level to present adjusted count per 1,000 people in each quantile group. The calculations are presented in Appendix 2.

Figure A.1.2 shows trends in amenable mortality for each quintile group by age and sex.

Figure A.1.2: Trends in amenable mortality (fixed x-axis for age group comparisons, then floating x-axis for deprivation quintile group comparisons)

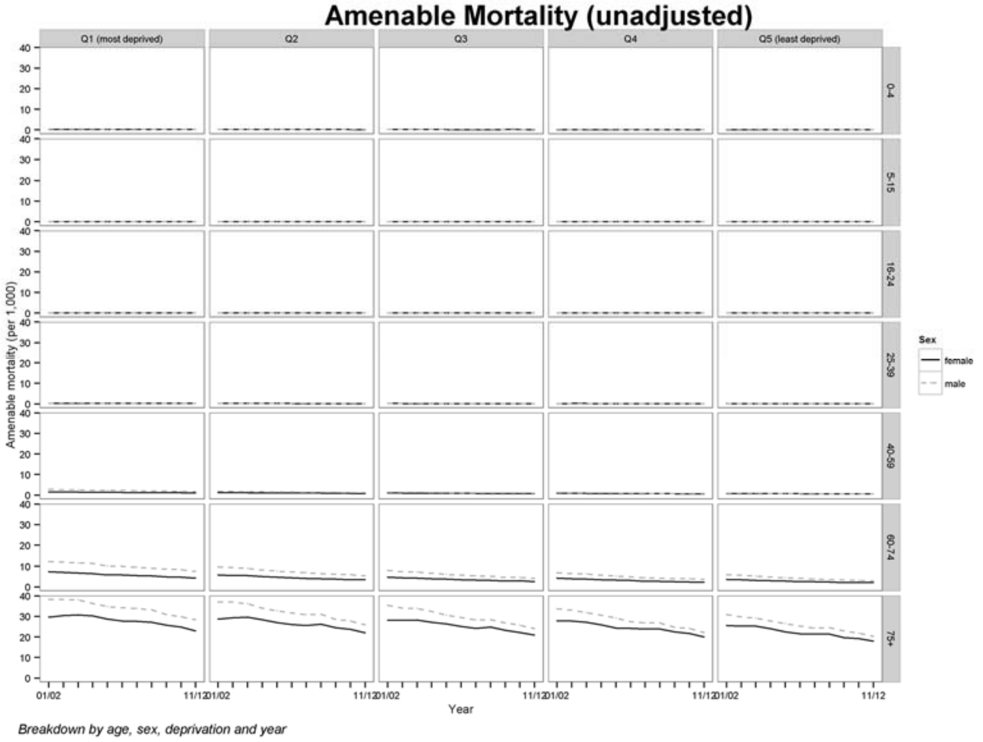

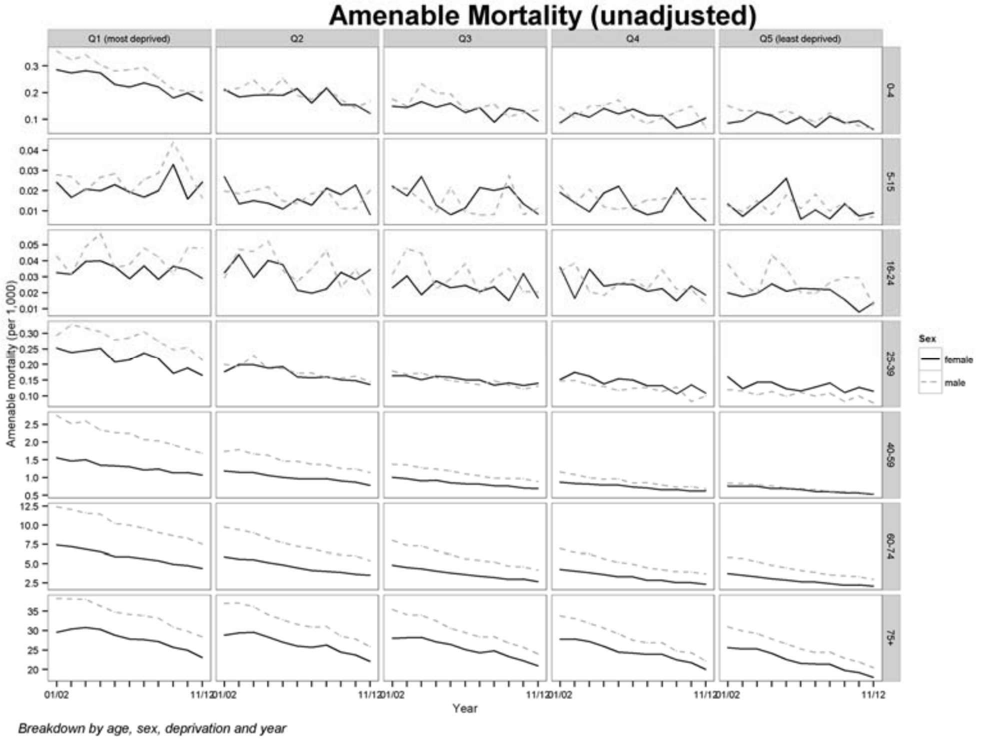

Table A.1.3: ONS list of causes of death considered amenable to health care (Office of National Statistics 2015)

Note: ONS produce separate lists for "amenable" and "preventable" deaths, where the latter are considered preventable by wider public health activities outside the health care system. In line with the NHS Outcomes Framework, we use the former list i.e. "amenable".

Condition group and cause	ICD-10 codes	Age
**Infections**		
Tuberculosis	A15-A19, B90	0-74
Selected invasive bacterial and protozoal infections	A38-A41, A46, A48.1, B50-B54, G00, G03, J02, L03	0-74
Hepatitis C	B17.1, B18.2	0-74
HIV/AIDS	B20-B24	All
**Neoplasms**		
Malignant neoplasm of colon and rectum	C18-C21	0-74
Malignant melanoma of skin	C43	0-74
Mesothelioma	C45	0-74
Malignant neoplasm of breast	C50	0-74
Malignant neoplasm of cervix uteri	C53	0-74
Malignant neoplasm of bladder	C67	0-74
Malignant neoplasm of thyroid gland	C73	0-74
Hodgkin's disease	C81	0-74
Leukaemia	C91, C92.0	0-44
Benign neoplasms	D10-D36	0-74
**Nutritional, endocrine and metabolic**		
Disorders of thyroid gland	E00–E07	0–74
Diabetes mellitus	E10-E14	0-49
**Neurological disorders**		
Epilepsy and status epilepticus	G40-G41	0-74
**Cardiovascular diseases**		
Rheumatic and other valvular heart disease	I01-I09	0-74
Hypertensive diseases	I10-I15	0-74
Ischaemic heart disease	I20-I25	0-74
Cerebrovascular diseases	I60-I69	0-74
**Respiratory diseases**		
Influenza (including swine flu)	J09-J11	0-74
Pneumonia	J12-J18	0-74
Asthma	J45-J46	0-74
**Digestive disorders**		
Gastric and duodenal ulcer	K25-K28	0-74

Acute abdomen, appendicitis, intestinal obstruction, cholecystitis/lithiasis, pancreatitis, hernia	K35-K38, K40-K46, K80-K83, K85, K86.1-K86.9, K91.5	0-74
**Genitourinary disorders**		
Nephritis and nephrosis	N00-N07, N17-N19, N25-N27	0-74
Obstructive uropathy and prostatic hyperplasia	N13, N20-N21, N35, N40, N99.1	0-74
**Maternal and infant**		
Complications of perinatal period	P00-P96, A33	All
Congenital malformations, deformations and chromosomal anomalies	Q00-Q99	0-74
**Injuries**		
Misadventures to patients during surgical and medical care	Y60-Y69, Y83-Y84	All

References

1. Formula Review Group. Review of the General Medical Services global sum formula. London: NHS Employers and BMA, 2007.

2. Hippisley-Cox J, Coupland C, Pringle M, et al. GMS Formula Review Analysis of the QRESEARCH database. Final Report to the GMS Review Group. Nottingham: NHS Employers, 2006.

3. Ashworth, M., Schofield, P., Doran, T., Cookson, R., Sutton, M., Seed, P. T & Fleetcroft, R. (2013). The Public Health Impact score: a new measure of public health effectiveness for general practices in England. British Journal of General Practice, 63(609), e291-e299.

4. Health and Social Care Information Centre. NHS Outcomes Framework 2014/15. Domain 2: Enhancing quality of life for people with long-term conditions. Clinical Indicators Team, 2014.

5. Office of National Statistics. Statistical Bulletin: Avoidable Mortality in England and Wales, 2013. ONS, 2015.
