# Supplementary material for: How a universal health system reduces inequalities: lessons from England
Source: J Epidemiol Community Health. 2016 Jan 19;70(7):637–43. doi: 10.1136/jech-2015-206742 (PMC4941190; doi:10.1136/jech-2015-206742)
Supplement: Web appendix 2 [file jech-2015-206742-s2.pdf]

# Appendix 2: Indirect age, sex standarisation of indicators at LSOA level

The indirect age sex standardisation process used to adjust the preventable hospitalisation and amenable mortality indicators:

$$adjusted\_count_{lsoa} = adjusted\_rate_{lsoa} \times population_{lsoa} \tag{1}$$

$$adjusted\_rate_{lsoa} = \frac{observed_{lsoa}}{expected_{lsoa}} \times rate_{national} \tag{2}$$

$$observed_{lsoa} = \sum^{sex}\sum^{age} events_{lsoa,age,sex} \tag{3}$$

$$expected_{lsoa} = \sum^{sex}\sum^{age} expected_{lsoa,age,sex} \tag{4}$$

$$expected_{lsoa,age,sex} = rate_{national,age,sex} \times population_{lsoa,age,sex} \tag{5}$$

$$rate_{national,age,sex} = \frac{\sum^{lsoa} events_{lsoa,age,sex}}{\sum^{lsoa} population_{lsoa,age,sex}} \tag{6}$$

$$rate_{national} = \frac{\sum^{lsoa}\sum^{sex}\sum^{age} events_{lsoa,age,sex}}{\sum^{lsoa}\sum^{sex}\sum^{age} population_{lsoa,age,sex}} \tag{7}$$

$$population_{lsoa} = \sum^{sex}\sum^{age} population_{lsoa,age,sex} \tag{8}$$

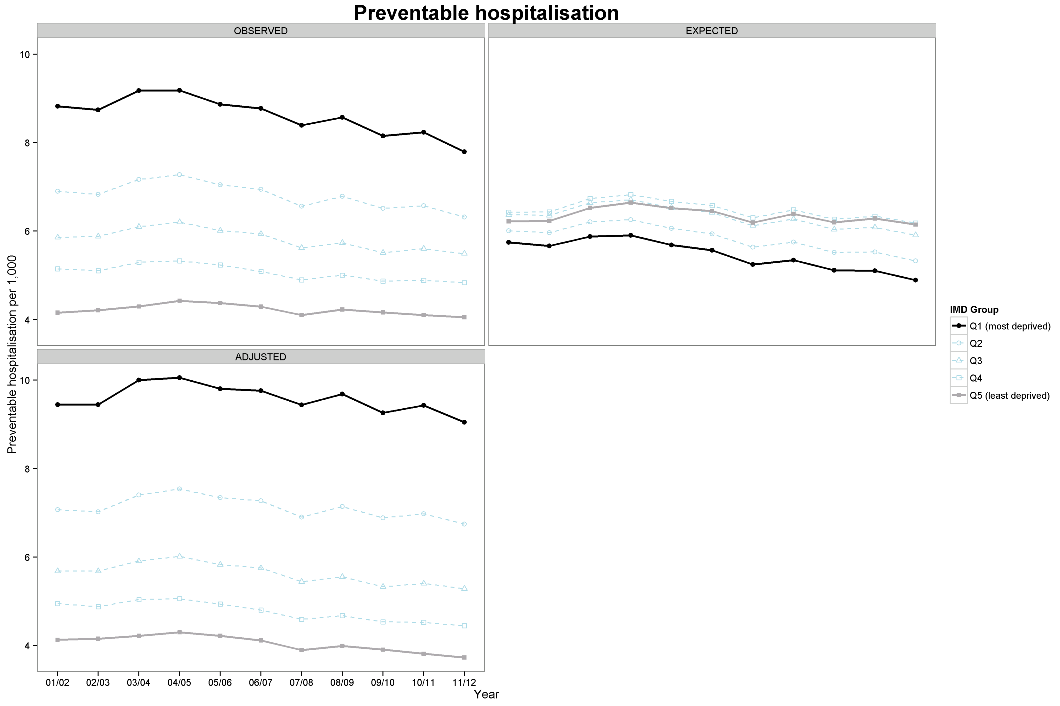


Figure 1: Preventable hospitalisation per 1,000 populations

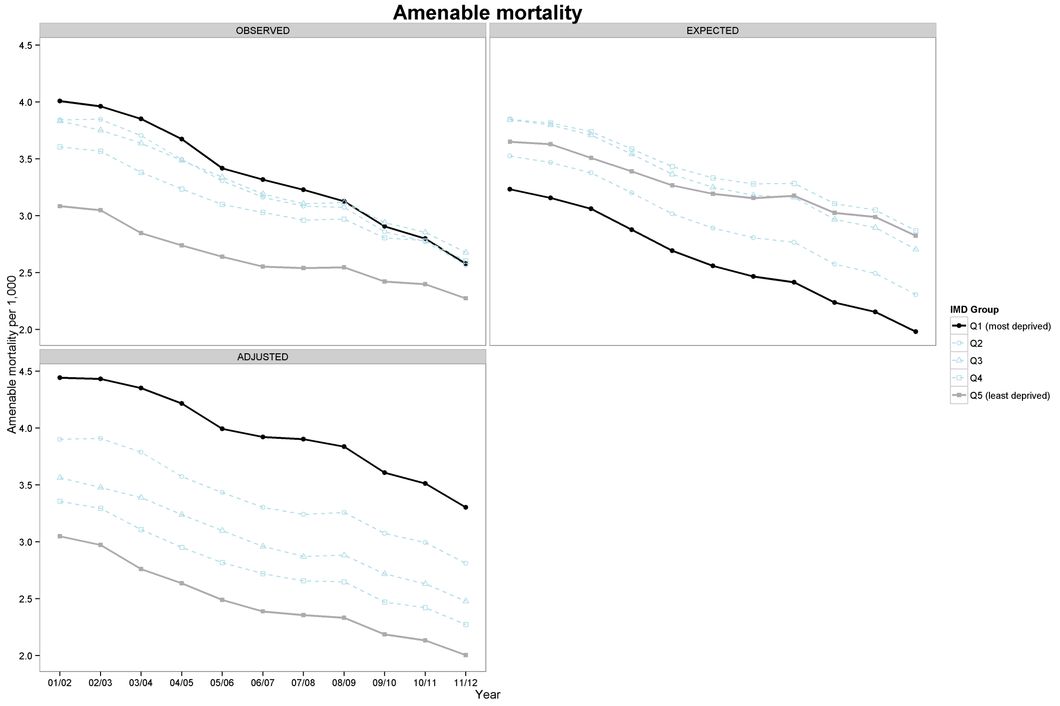


Figure 2: Amenable mortality per 1,000 population
