# Supplementary material for: How a universal health system reduces inequalities: lessons from England
Source: J Epidemiol Community Health. 2016 Jan 19;70(7):637–43. doi: 10.1136/jech-2015-206742 (PMC4941190; doi:10.1136/jech-2015-206742)
Supplement: Web appendix 3 [file jech-2015-206742-s3.pdf]

Appendix 3: Robustness checks

Exception reporting

Figure A.3.1 shows trends in primary care quality in terms of public health impact score, relative index of inequality and slope index of inequality. The base case analysis shown in the top half of the panel was conducted using reported achievement, which excludes "exception reported" patients from the population denominator, the bottom half of the panel reports population achievement, which includes exceptions as poor quality. Data on "exception reported" patients was not available in the first year 2004/5 and so in the population achievement series average primary care quality for that year is artificially inflated, since "exception reported" patients could not be included in that year whereas in all other years "exception reported" patients were included as poor quality. As we can see despite the different absolute levels of performance as calculated by the two alternative calculation methods for this indicator the inequality trends in both absolute and relative terms are almost identical for the two alternative measures.

Figure A.3.1: Trends in Primary Care Quality

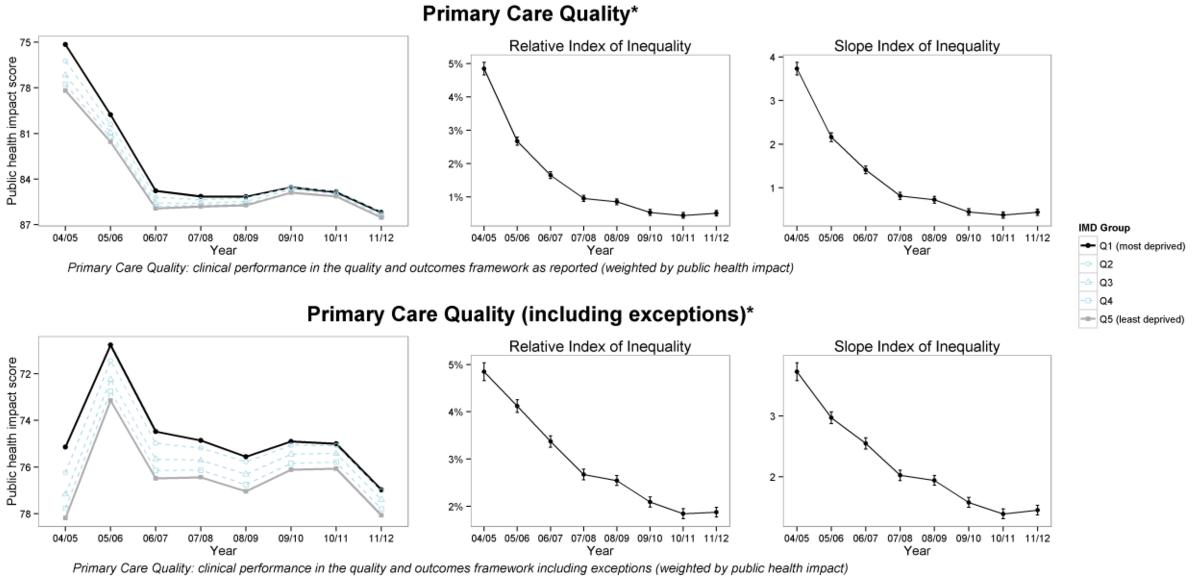

Change in neighbourhood deprivation

We used IMD 2010 (published in 2010 using data relating to 2007) for all years to ensure that our findings reflected real changes in health care delivery and outcomes, rather than artificial changes in the calculation of the deprivation index or the composition of neighbourhoods. This does raise the issue, however, of how accurately the deprivation of a neighbourhood in 2007 reflects its deprivation in 2004/5 and 2011/12. To assess this, we looked at cross tabulations of change over the seven year period between IMD 2004 (data for 2001) and IMD 2010 (data for 2007). These show that 84% of LSOAs in the most deprived fifth remained in the most deprived fifth, that 88% of neighbourhoods in the least deprived fifth remained in the least deprived fifth, and that only 14% of LSOAs changed rank by the equivalent of one quintile group or more.
